# Effect of Ginkgo biloba extract on experimental cardiac remodeling

**DOI:** 10.1186/s12906-015-0719-z

**Published:** 2015-08-13

**Authors:** Wei Li, Zhenhua Luo, Xingde Liu, Lingyun Fu, Yini Xu, Lirong Wu, Xianchun Shen

**Affiliations:** Department of Cadiovascular Medicine, Affiliated Hospital of Guizhou Medical University, Beijing Road, , Guizhou Guiyang, 550004 China; Central laboratory, Guizhou Provincial People’s Hospital, Zhongshan East Road 83, Guiyang, 550002 Guizhou China; Key Laboratory of Optimal utilization of Natural Medicinal Resources, School of Pharmaceutic Science, Guizhou Medical University, Huaxi College Station, Guian New District, 550025 Guizhou China

**Keywords:** *Ginkgo biloba* extract, Acute myocardial infarction, TGF-β1, Type I collagen, Matrix metallopeptidase 2, Matrix metallopeptidase 9

## Abstract

**Background:**

To investigate the ameliorated effects of an extract of *Ginkgo biloba* extract (GBE) on experimental cardiac remodeling in rats induced by acute cardiac infarction, and further explore the mechanism concentrated on myocardial type I collagen, transforming growth factor beta 1 (TGF-β1), matrix metalloproteinase 2 (MMP-2) and matrix metalloproteinase 9 (MMP-9), and provide the experimentaldata for clinical application of GBE.

**Methods:**

Rats were divided into five groups (*n* = 20) as following: sham operation group (group A), acute myocardial infarction model group (group B), acute myocardial infarction model + aspirin (10 mg/kg) treatment group (group C), acute myocardial infarction model + captopril (20 mg/kg) treatment group (group D) and acute myocardial infarction model + *Ginkgo biloba* extract (100 mg/kg) treatment group (group E). The rat acute myocardial infarction model was reproduced by ligaturing the left anterior descending artery excluding the sham operation group which did not ligation only completed the operational process. Each group was further subdivided into treatment regimens lasting 4 weeks and 8 weeks. Immunohistochemistry and real-time polymerase chain reaction (PCR) methods were used to detect the protein expression and mRNA transcriptional levels of rat myocardial TGF-β1, type I collagen, MMP-2 and MMP-9.

**Results:**

Compared with group B, regardless of the length of treatment (4 or 8 weeks), the TGF-β1, MMP-2 and MMP-9 mRNA transcriptional levels, and the protein expression levels of type I collagen, MMP-2 and MMP-9 in groups D, C and E were significantly decreased (*P* < 0.01). Furthermore, the mRNA expression levels of TGF-β1 in groups D, C and E were significantly lower after 8 weeks compared to after 4 weeks (*P* < 0.01), as were the expression levels of type I collagen in groups D, C and E (*P* < 0.05). There was no statistically significant difference in the protein expression levels of MMP-2 and MMP-9 between groups E and C.

**Conclusions:**

GBE could inhibit experimental rat myocardial remodeling after acute myocardial infarction via reduced transcription of TGF-β1, MMP-2 and MMP-9 genes and by the decreased expression of type I collagen, MMP-2 and MMP-9 proteins in myocardial cells.

**Electronic supplementary material:**

The online version of this article (doi:10.1186/s12906-015-0719-z) contains supplementary material, which is available to authorized users.

## Background

Cardiac ventricular remodeling can lead to ventricular dilatation, heart function disorders, chronic heart failure, and even sudden death in patients [1]. Previous accumulated evidences had confirmed to the generation of myocardial fibrosis, the abnormal metabolism of collagen and extracellular matrix (ECM) remodeling after AMI, as well as the association of other factors involved in the process of cardiac remodeling [2–4]. The infarct scar, formed by myocardial remodeling, can result in cardiac systolic and diastolic dysfunction, myocardial hypertrophy, an increase of the ECM at the site of the myocardial infarction or pseudo-infarction, myocardial fibrosis, etc., which ultimately lead to heart failure [5]. Therefore, finding a reasonable and effective drug to prevent and reverse cardiac remodeling is of great significance to reduce AMI mortality and has become a highlighted point of pharmaceutical research.

Ginkgo Folium, Chinese name Yinxingye, had widely used in the world for treated cardiovascular diseases. *Ginkgo biloba* extracts (GBEs) was prepared from Ginkgo Folium, included the flavonoids and terpene lactone compounds, which is the mainly bioactive ingredients for cardiovascular diseases [6]. GBEs protected against myocardial ischemic injury, the mechanism of which may be associated to the scavenging of free radicals and anti-lipid peroxidation, consequently dilated coronary blood vessels, ameliorated microcirculation and inhibited cardiac myocytes apoptosis [7, 8]. Recently data confirmed that GBEs had a significant reversing effect on liver and kidney fibrosis [9]. Previously, Liu et al. [10] reported that GBE (nam ed GBE50) could inhibit the abnormal expression of the Na^+^–Ca^2+^ exchanger in rat myocardial cells after AMI, thereby improving and recovering systolic and diastolic function and preventing ischemic injury. However, the inhibiting or reversing effects of GBEs on cardiac remodeling after AMI have rarely been reported. In order to investigate the preventive and therapeutic effects of GBEs on cardiac remodeling, we observed the changes in type I collagen, transforming growth factor β1 (TGF-β1) and matrix metalloproteinases 2 and 9 (MMP-2 and MMP-9, respectively) associated to myocardial remodeling after AMI in rats treatment with GBE.

## Methods

### Materials

GBE (batch number: WGBEXP130918) was provided by researchers at the Guizhou Provincial Biochemical Engineering Center as a yellow powder. The bioactive ingredients of the GBE extract were included 26.16 % Ginkgo favonelucosides (quercetin 11.06 %, kaempferol 11.93 % and isorhamnetin 3.17 %) and 6.28 % total terpene lactone (ginkgolide C 1.23 %, bilobalide 1.81 %, ginkgolide A 2.48 % and ginkgolide B 0.76 %) (Additional file 1). The content of Ginkgaolic acid was less than 1PPM. Aspirin (asp) and chloral hydrate were obtained from Shanghai Aladdin Reagent Company. Captopril (cap) was obtained from HuBei-Sihuan Pharmaceuticals Co., Ltd. Type I collagen antibody was obtained from Abcam, UK, while MMP-2 and 9 antibodies (rabbit anti-mouse polyclonal antibodies), and the goat anti-rabbit streptavidin–biotin complex (SABC) secondary antibody immunohistochemical kit were obtained from Wuhan Boster Biological Engineering Co., Ltd. 3,3′-Diaminobenzidine (DAB) was from Beijing Zhongshan Golden Bridge Biotechnology Co. Ltd, and RNAiso (a total RNA extraction reagent) and agarose were from Sigma, USA. Finally, RNA-EZ reagent D (an RNA protective agent), the RevertAid First Strand cDNA Synthesis Kit and SYBR® Premix Ex Taq™ II were obtained from Takara Biotechnology, Inc.

### Animals

Male adult Sprague–Dawley (SD) rats, weighing 200–250 g, were purchased from the Experimental Animal Center, Daping Hospital, The Third Military Medical University. The animals were housed in the Experimental Animal Center, Guizhou Medical University under specified pathogen-free (SPF) conditions with a 12-hour light/dark cycle and free access to food and water. All experimental procedures were performed in accordance with the guidelines of the Experimental Animal Care and Institutional Animal Ethical Committee of Guizhou Medical University (certificate number: SCXK2012-0001).

### AMI model in rats

SD male rats, weighing 200 ~ 250 g, were reproduced the myocardial infarction model by ligated the left anterior descending artery as previous methods [11]. Rats were treated with 10 % chloral hydrate solution (0.3 mL/100 g) intraperitoneally for general anesthesia prior to surgery. A small animal ventilator was connected to rats during surgery (HX-300, Chengdu TME Technology Co., Ltd) and the electrocardiograms showed arched elevations in the ST segment (6851-K-type ECG machine, Hitachi, Ltd), indicating the successful establishment of the model. According to preliminary experiments, the appropriate drug dosage was designed to be 100 mg/kg GBE, 10 mg/kg asp and 20 mg/kg cap. The model rats were randomly divided into 4 groups as follows: an AMI model group (group B), an AMI + asp group (group C), an AMI + cap group (group D) and an AMI + GBE group (group E), with 20 rats per group. A sham operation group (group A) was not ligated the artery only completed operation process. Each drug was given 24 h after the successful establishment of the AMI model with an administration volume of 1 mL/100 g via gavage once daily, respectively. Rats in groups A and B were administered the same volume of sterile distilled water once daily. After treatment for 4 and 8 weeks, 10 % chloral hydrate 0.4 mL/100 g was given intraperitoneally and rats were sacrificed. Myocardial tissue was extracted and placed in ice-cold TRIzol® (5 mL) before storing at −80 °C.

### Myocardial tissue immunohistochemistry

Myocardial tissue was fixed in 10 % formalin for 3 days and then dehydrated through gradient alcohols for 30 min, followed by a solution of benzene/ethanol (1:1 *v*/*v*) for 2 × 30 min and then benzene transparency for 30 min. The tissue was then treated with benzene/ paraffin wax (1:1 *v*/*v*) for 30 min and then waxdip for 50 min. Slices were prepared with a vibrating blade microtome (Leica VT1000 S, Germany), with five slices prepared for each tissue. Samples were then treated with a solution of hydrogen peroxide (H_2_O_2_, 30 %) and distilled water (1:10 *v*/*v*) in the dark at room temperature for 5 ~ 10 min to inactivate endogenous enzymes, before washing three times with distilled water. The sections were then immersed in 0.01 M citrate buffer (pH 6.0), heated to boiling and washed with phosphate-buffered saline (PBS, pH 7.2 ~ 7.6, 1–2 times) after cooling for antigen retrieval. Bovine serum albumin (BSA, 10 %) was used to close the antigen by incubating at room temperature for 30 min. Rabbit anti-mouse type I collagen primary antibody (1:400, 30 μL/piece), MMP-2 primary antibody (1:100, 50 μL/piece) and MMP-9 primary antibody (1:100, 50 μL/piece) were added, followed by incubation at 37 °C for 60 min. Secondary antibody was added after washing with PBS and samples were then incubated at 37 °C for 50 min and washed with PBS before the addition of DAB for 3 ~ 5 min. Samples were washed with distilled water after hematoxylin counterstaining for 45 s and then dehydrated through graded alcohols and steeped in xylene for 35 min. Neutral gum was used for mounting. Brown granules in the cells were observed by microscopy (Olympus, Japan), indicating a positive result. A Biomias2001 color high-definition image analysis system was used for analysis and observation, and 10 fields (400×) were randomly selected in each slice to calculate the average gray scale (AU). PBS was taken as a negative control.

### Real-time polymerase chain reaction (PCR) analysis

RNA was extracted from 100 mg myocardial tissue and homogenized by the addition of 800 μL TRIzol® diluted in chloroform (20:1 *v*/*v*) with vortexing for 15 s, followed by centrifugation at 4 °C, 12,000 rpm for 15 min (TGLL-18G high-speed refrigerated centrifuge, Eppendorf). Isopropanol was then added to precipitate the RNA (1:2 *v*/*v*), followed by incubation at room temperature for 10 min and centrifugation at 4 °C, 12,000 rpm for 10 min. The supernatant was discarded and the RNA was washed with 1 mL 75 % ethanol, followed by centrifugation at 4 °C, 7500 rpm for 5 min. The supernatant was again discarded and the resulting RNA pellet was dried at room temperature for 5 ~ 10 min before the addition of diethylpyrocarbonate (DEPC)-treated water. UV spectrophotometry (BIOTEK™ Corporation, USA) was used to detect the RNA concentration and purity.

Reverse transcription of the cDNA was achieved with a TaKaRa reverse transcription kit according to the manufacturer’s instructions. All operations were carried out on ice and the reaction system was 5× gDNA eraser buffer (2 μL), gDNA eraser (1 μL) and total RNA (1 μL, concentration ≤ 1000 ng). RNase-free dH_2_O was added to achieve a volume of 10 μL. The reaction mixture was incubated at 42 °C for 2 min and then the reaction solution was added to the following system: 5× PrimeScript buffer (4.0 μL), RT enzyme mix I (1.0 μL) and 4× RT primer mix (1.0 μL). RNase-free dH_2_O was added to achieve a final volume of 40 μL. PCR amplification (Applied Biosystems PE9700, Perkin Elmer, USA) was conducted at 37 °C for 15 min and then 85 °C for 5 s. The amplified cDNA was stored at 4 °C. A fluorescent quantitative PCR instrument (Roche, Germany) was used for detection. Briefly, 20 μL of the aforementioned reaction solution in the reaction system was prepared on ice with 2× SYBR® Green Mix reagent, SYBR® Premix Ex Taq™ II (10 μL), the PCR forward primer (10 μm, 0.8 μL), the PCR reverse primer (10 μm, 0.8 μL), the DNA template (2 μL) and sterile ultrapure water (6.4 μL). The PCR reaction conditions were: 95 °C for 30 s, 95 °C for 5 s and 60 °C for 20 s for 40 cycles. The experiment was repeated three times with three replicates for each sample and sterile ultrapure water as a negative control. β-Actin was taken as an internal reference and the standardized 2^–△△*C*^_T_ method was used to analyze the mRNA expression levels of TGF-β1, MMP-2 and MMP-9 (the primers are shown in Table 1 and were synthesized by Shanghai Biological Engineering Technology Services Company) in rat myocardial tissue to conduct a relative quantitative analysis.

**Table 1:** Amplification primers used in real-time PCR

Gene	Primer sequence (5′ to 3′)	Gene sequence number
TGF-β1 F1	GCTCCTGTGACAGCAGGGAT	NM_000660.5
TGF-β1 R1	GGCAGAAGTTGGCATGGTAG	
MMP-2 F1	GTGCCAAGGTGGAAATCAGAG	NM_031054.2
MMP-2 R1	AAGGTTGAAGGAAACGAGCGA	
MMP-9 F1	GTCTTCCCCTTCGTCTTCCT	NM_031055.1
MMP-9 R1	ACCCCACTTCTTGTCAGCGT	

### Statistical analysis

Data for each set of experiments fitted a normal distribution after verification and the mean was expressed ± standard deviation ($\overline{\chi}\pm s$). The comparison of the differences between the groups was conducted using a group analysis of variance design, making pairwise comparisons when it was statistically significant. The data between week four and week eight were compared using a paired *t*-test. SPSS version 19.0 software was used for the statistical analysis, where *P* < 0.05 was considered statistically significant.

## Results

### Effect of GBE on myocardial type I collagen after AMI in rats

The mortality rates for the groups were as follows: one and two rats after 4 weeks and 8 weeks, respectively, in the sham group; two and three rats after 4 weeks and 8 weeks, respectively, in the AMI model group; three rats after 8 weeks in the AMI + asp group; two rats after 8 weeks in the AMI + cap group; and two rats after 8 weeks in the AMI + GBE group. The remaining rats were used in follow-up experiments.

**Fig. 1:**
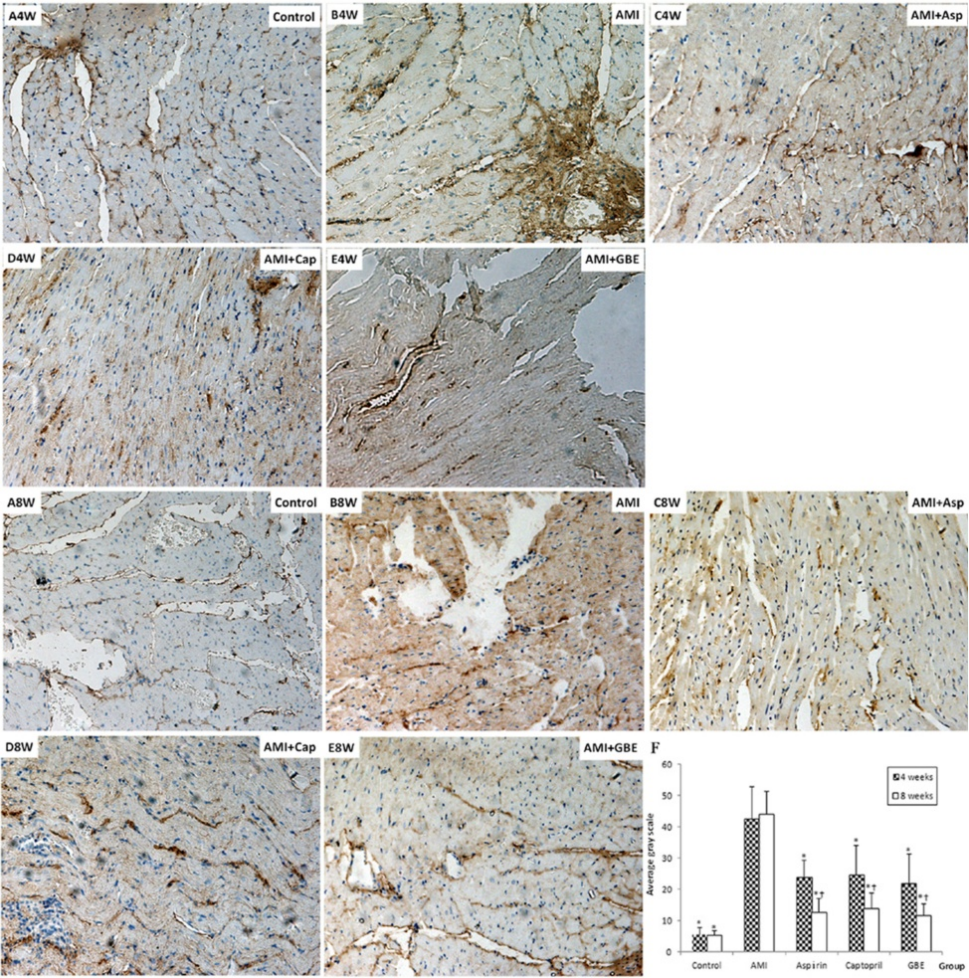
Immunohistochemical detection of myocardial type I collagen in rats treated with GBE, asp and cap for 4 and 8 weeks compared with sham and AMI model controls (200×). A-4 week and 8 Week: Sham-operated negative controls at 4 and 8 weeks, respectively; B-4 week and 8 week: AMI model controls at 4 and 8 weeks, respectively; C-4 week and 8 week: asp-treated AMI groups at 4 and 8 weeks, respectively; D-4 week and 8 week: cap-treated AMI groups at 4 and 8 weeks, respectively; E-4 week and 8 week: GBE-treated AMI groups at 4 and 8 weeks, respectively. “*” signifies *P* < 0.05 compared with the AMI model control; “†” signifies *P* < 0.05 compared with the same group at 8 and 4 weeks

Collagen I was expressed in the cytoplasm and immunopositive stained (brown) particles were located in the nuclear periplasm and its projections, and the depth of the brown coloring was representative of the expression level. After operation 4 and 8 weeks, the expression levels of collagen I in the AMI model group were significantly higher than that of the sham group (*P* < 0.01). The GBE-, asp- and cap-treated groups were significantly inhibited the collagen I expression level compared with model group (*P* < 0.01). At 8 weeks, the expression levels of myocardial type I collagen in GBE-, asp- and cap-treated groups were lower than at 4 weeks (*P* < 0.05). There was no statistically significant difference in the expression levels of type I collagen between the GBE-, asp- and cap-treated groups (Fig. 1).

### Effect of GBE on myocardial MMP-2 and MMP-9 proteins after AMI in rats

**Fig. 2:**
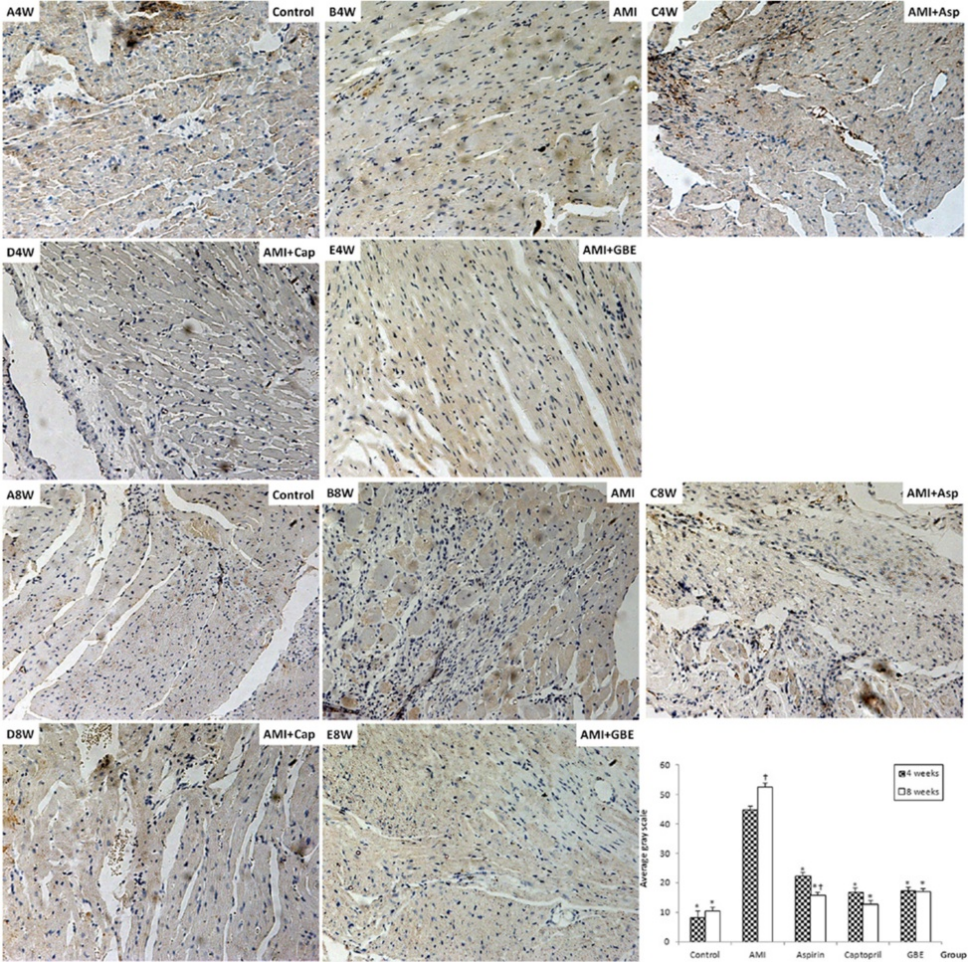
Immunohistochemical detection of myocardial MMP-2 protein in rats treated with GBE, asp and cap for 4 and 8 weeks compared with sham and AMI model controls (200 ×). A-4 week and 8 Week: Sham-operated negative controls at 4 and 8 weeks, respectively; B-4 week and 8 week: AMI model controls at 4 and 8 weeks, respectively; C-4 week and 8 week: asp-treated AMI groups at 4 and 8 weeks, respectively; D-4 week and 8 week: cap-treated AMI groups at 4 and 8 weeks, respectively; E-4 week and 8 week: GBE-treated AMI groups at 4 and 8 weeks, respectively. “*” signifies *P* < 0.05 compared with the AMI model control; “†” signifies *P* < 0.05 compared with the same group at 8 and 4 weeks

**Fig. 3:**
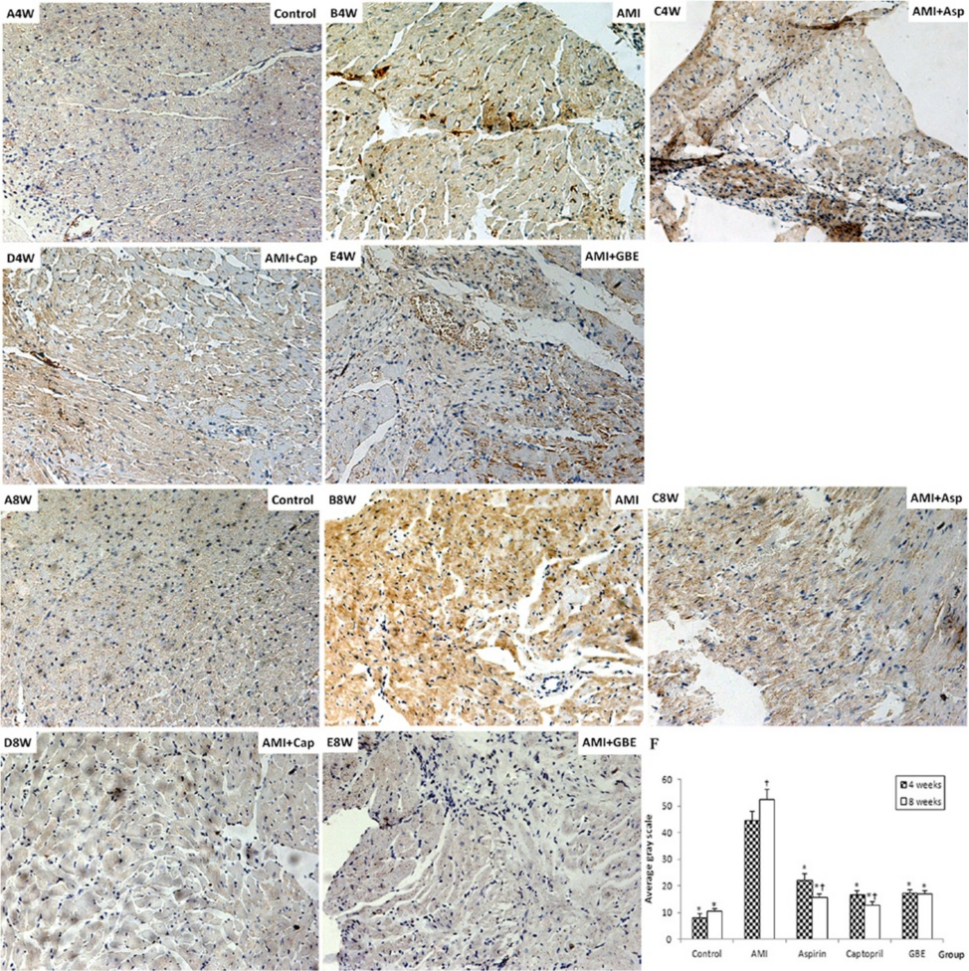
Immunohistochemical detection of myocardial MMP-9 protein in rats treated with GBE, asp and cap for 4 and 8 weeks compared with sham and AMI model controls (200 ×). A-4 week and 8 Week: Sham-operated negative controls at 4 and 8 weeks, respectively; B-4 week and 8 week: AMI model controls at 4 and 8 weeks, respectively; C-4 week and 8 week: asp-treated AMI groups at 4 and 8 weeks, respectively; D-4 week and 8 week: cap-treated AMI groups at 4 and 8 weeks, respectively; E-4 week and 8 week: GBE-treated AMI groups at 4 and 8 weeks, respectively. “*” signifies *P* < 0.05 compared with the AMI model control; “†” signifies *P* < 0.05 compared with the same group at 8 and 4 weeks

At present, the MMP-2 and MMP-9 proteins were detected by immunohistochemical methods. The expression levels of MMP-2 and MMP-9 proteins in the model group were higher than that in the sham group (*P* < 0.05) after AMI 4 and 8 weeks. After treated with GBE-, asp- and cap attenuated the MMP-2 and MMP-9 proteins levels, there was significantly difference compared with model group (*P* < 0.05). The expression levels of MMP-2 and MMP-9 proteins in model group at 8 weeks were higher than at 4 weeks (*P* < 0.05). However, at 8 weeks the expression level of MMP-2 in the asp-treated group and the expression levels of MMP-9 in the asp- and cap-treated groups were significantly lower than those at 4 weeks (*P* < 0.05). At 4 weeks the protein expression levels of MMP-2 and MMP-9 in the GBE-treated group were significantly lower than those for the asp-treated group (*P* < 0.01); however, at 8 weeks there was no significant difference in the expression levels of MMP-2 and MMP-9 proteins between the GBE-treated group and the asp-treated group (Figs. 2 and 3).

### Effect of GBE on TGF-β1 mRNA transcription after AMI in rats

**Fig. 4:**
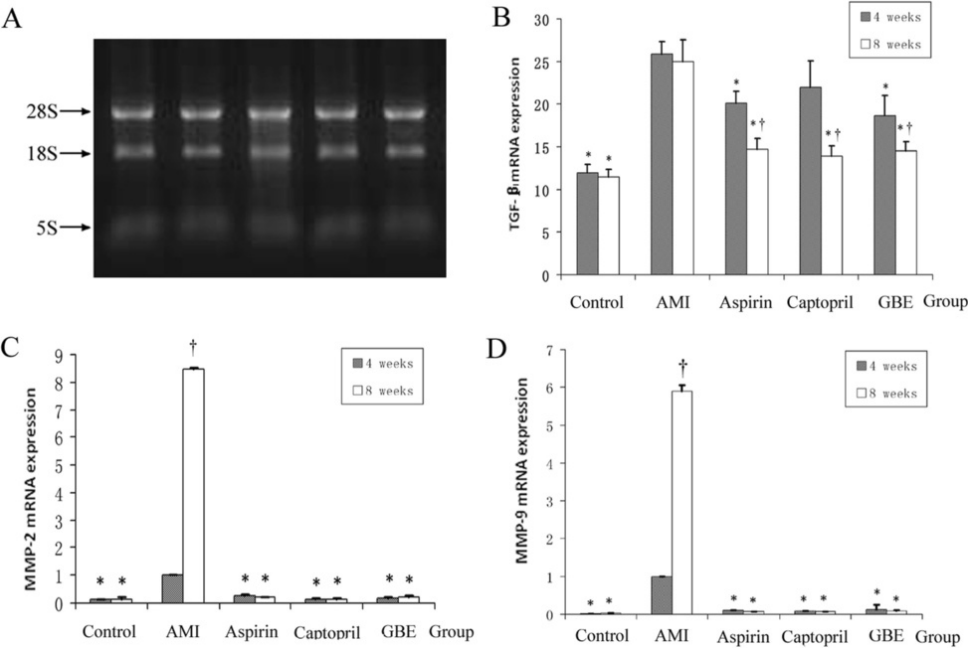
mRNA expression levels of myocardial type I collagen, MMP-2 and MMP-9 in rats treated with GBE, asp or cap for 4 and 8 weeks compared with sham and AMI model controls. **a** Myocardial tissue-extracted RNA electrophoresis pattern; **b** type I collagen mRNA expression in each group; **c** MMP-2 mRNA expression in each group; **d** MMP-9 mRNA expression in each group. “*” signifies *P* < 0.05 compared with the AMI model group; “†” signifies *P* < 0.05 compared with the same group at 8 and 4 weeks

RNA electrophoretogram indicated that three bands for 28S RNA, 18S RNA and 5S RNA in rats and the brightness ratio of 28S RNA and 18S RNA was about 2:1, while the ratio of the optical densities (ODs) at 260 nm and 280 nm (OD260/OD280) was between 1.9 and 2.1, which indicated the RNA could be used for subsequent RT-PCR experiments (Fig. 4a).

At 4 and 8 weeks, the mRNA expression levels of TGF-β1 in the AMI model group were significantly higher than in the sham group (*P* < 0.01). In addition, compared with the AMI model group, the mRNA expression levels of myocardial TGF-β1 in the GBE-, asp-treated groups were significantly alleviated (*P* < 0.01). Furthermore, at 8 weeks the mRNA expression levels of TGF-β1 in the GBE-, asp- and cap-treated groups were significantly less than at 4 weeks (*P* < 0.01) (Fig. 4b).

### Effect of GBE on mRNA transcription levels of MMP-2 and MMP-9

After AMI 4 and 8 weeks, the mRNA levels of MMP-2 and MMP-9 in model were significantly higher than in the sham group (*P* < 0.05). Administrated with GBE, asp, and cap could alleviated the MMP-2 and MMP-9 mRNA expression increase (*P* < 0.05). Furthermore, the mRNA expression of MMP-2 and MMP-9 in the AMI model group at 4 weeks was significantly lower than at 8 weeks, however there was no statistically significant difference in the mRNA expression levels of the other groups between weeks 4 and 8 weeks (Fig. 4c and d).

## Discussion

Cardiovascular disease accounts for nearly 40 % of all deaths annually in developed countries. AMI is the leading cause of congestive heart failure and death in industrialized world. Myocardial infarction (MI) often leads to adverse ventricular remodeling resulting in changes involving the size, shape, and function of heart, and the subsequent development of heart failure [1, 12]. Ventricular remodeling is changes in cell structure and function, such as myocardial fibrosis, which can be caused by a series of complex molecular and cellular mechanisms, such as metabolic abnormalities of the interstitial collagen network, changes in the TGF-β molecular pathways and changes in the MMPs [13, 14]. Inhibiting or blocking the various key pathological factors induced ventricular remodeling could effectively prevent and reverse the development of cardiac remodeling [15]. GBEs, extract from the leaves of the gingko tree, are widely applied in cardiovascular disease which is mainly flavonoids and terpene lactones, as well as other compounds that are known to play a role in the therapeutic treatment of cardiovascular and cerebrovascular diseases [16]. At present study, the present results confirmed that GBEs could ameliorate ventricular remodeling induced by AMI.

TGF-β1, the most significant cytokine that induces fibrosis in vivo, can stimulate the synthesis of collagen, fibronectin, proteoglycan and other intercellular substances, and then leads to an increased expression of type I/III collagen. Plenty of evidences confirmed that over-expression TGF-β1 had become a common pathway for different pathological factors leading to myocardial fibrosis [17]. Recently, TGF-β1 had been highlighted on preventing or reversing organ fibrosis via the TGF-β/Smads signaling system [18]. At present study, our results indicated that 100 mg/kg GBE can efficiently attenuate the mRNA expression of TGF-β1 after AMI 4 weeks, with significantly lower levels after 8 weeks, suggesting that GBE could decrease the expression of myocardial TGF-β1 in AMI rats in a time-dependent manner. Further, we speculated that GBE may decrease the expression of collagen by reducing TGF-β1 expression, thereby inhibiting the development of fibrosis.

It is well known that there are five type myocardial collagens, where collagen I and III are the main types found in the heart. When the ratio of collagen is disbalanced (i.e., the ratio of type I to type III is increased), decreases the ventricular wall compliance, increases stiffness, and diastolic filling is limited. Finally, myocardial contractile function also becomes limited [17]. Some researchers have found that *Salvia miltiorrhiza* can reduce myocardial type I collagen, while the flavonoids in *Astragalus membranaceus* have been found to improve hemodynamics after myocardial infarction, suggesting that some traditional Chinese medicines may inhibit cardiac remodeling by reducing the content of myocardial type I collagen [19–21]. This study found that GBE could effectively alleviate the expression of type I collagen in rats after AMI with time-dependent manner, GBE may inhibit myocardial remodeling after AMI by decreasing the expression of type I collagen.

Matrix metalloproteinases, a family of important zinc-dependent proteolytic enzymes, specifically hydrolyzed the ECM, play an important role in pathological remodeling. Shimizu et al. [22] found that MMP-2 mRNA in the non-infarcted zone of the rat left ventricular was 1.4 times higher than the control group, and the infarct zone of the left ventricular was increased 6.3 times. Meanwhile, immunofluorescence analysis found that there were obvious markers for MMP-2 in cardiomyocytes and the sarcolemma, suggesting that MMP-2 participates in ventricular remodeling through the synthesis and secretion of MMPs. This study showed that the mRNA expression levels of MMP-2 and MMP-9 proteins in cardiomyocytes after AMI in rats were significantly higher compared to sham-operated rats. Furthermore, the expression levels increased with time, suggesting that MMP-2 and MMP-9 are involved in the process of myocardial remodeling after AMI in rats.

Cap, a synthetic non-peptide angiotensin-converting enzyme (ACE) inhibitor, mainly affects the renin–angiotensin–aldosterone (RAA) system. This study found that cap had a significant inhibiting effect on myocardial MMP-2 and MMP-9 expression after AMI and could effectively delay cardiac remodeling [23, 24].

As an anti-inflammatory drug, asp has been established as a classic medication for the treatment of AMI, unstable angina and the secondary prevention of AMI. Kalkman et al. [25] found that asp has an inhibiting effect on MMP-9 in nasal polyps and can reduce the expression of nuclear factor kappa-light-chain-enhancer of activated B cells (NF-κB), cyclo-oxygenase 2 (COX-2), TGF-β and other factors by directly inhibiting the TGF-β/Smads signaling system, thereby reducing inflammation and fibrosis, and delaying cardiac remodeling [26]. In this study, cap and asp were established as positive controls and the results showed that GBE could significantly inhibit the mRNA and protein expression levels of myocardial remodeling-related enzymes MMP-2 and MMP-9 in the myocardium, suggesting that GBE may reduce ventricular remodeling after AMI in rats to a certain extent. This provides a new perspective for drug research focused on ventricular remodeling after AMI and suggests a role for herbal medicines, such as GBE.

This study found that GBE could significantly decrease the expression levels of myocardial TGF-β1 and type I collagen in AMI rats, but it was unclear whether it was acting by inhibiting myocardial TGF-β1 to decrease the expression of type I collagen or by inhibiting type I collagen to decrease the expression of TGF-β1. Studies have shown that, after myocardial infarction, the expressions of TGF-β1 and type I collagen are positively correlated. After myocardial infarction, TGF-β1 significantly increases and acts on the transcription and translation of collagen, inducing the production of procollagen mRNA, thereby further promoting the formation and deposition of collagen [27, 28] and promoting the synthesis of cardiac fibroblast type I collagen. This suggests that GBE may reduce the expression of type I collagen via inhibition of the myocardial TGF-β pathway in AMI rats. However, the specific mechanism remains to be further studied.

## Conclusion

The present results confirmed that that GBE could ameliorate the cardiac remodeling induced by AMI, the mechanism was partly involved in regulating expression of TGF-β1, MMP-2 and MMP-9, and finally attenuating the extracellular matrix deposition.

## Additional file

Additional file 1: Figure S1. The ingredintes of GBE.
