# Supplementary material for: Effect of Ginkgo biloba extract on experimental cardiac remodeling
Source: BMC Complement Altern Med. 2015 Aug 13;15:277. doi: 10.1186/s12906-015-0719-z (PMC4534054; doi:10.1186/s12906-015-0719-z)
Supplement: Additional file 1: Figure S1. — The ingredintes of GBE. [file 12906_2015_719_MOESM1_ESM.doc]

Figure 1:The active ingredients of Ginkgo biloba extract (batch number: WGBEXP130918 ) identified by Agilent 1100 HPLC

**
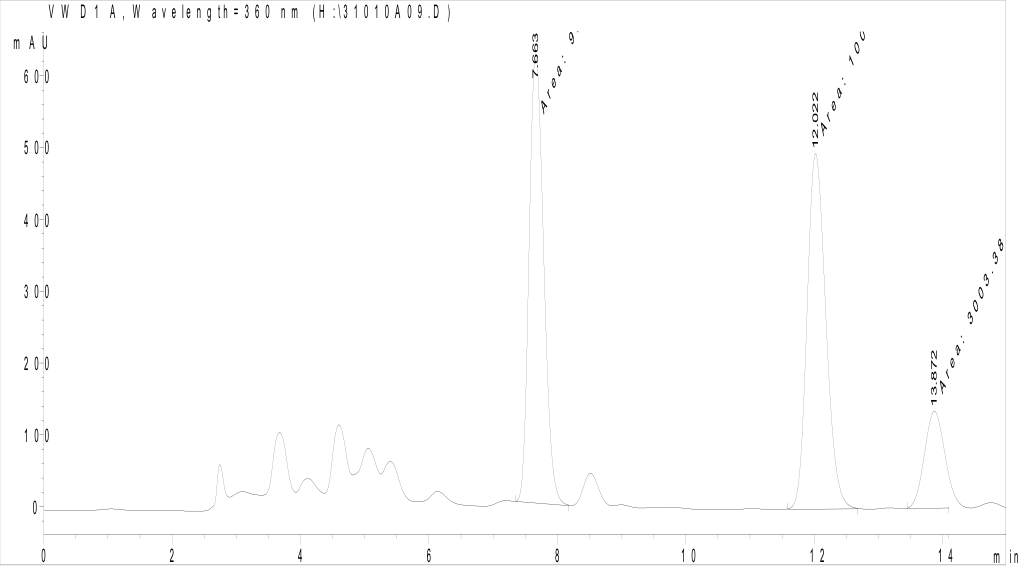
**

| Ingredient | Retention time | peak area | component |
| --- | --- | --- | --- |
| quercetin | 7.66 | 9912 | 11.06% |
| kaempferol | 12.02 | 10097 | 11.93% |
| isorhamnetin | 13.87 | 3003 | 3.17% |
| Ginkgo favone lucosides | --- | ---- | 26.16% |

Figure 2: The active ingredients of Ginkgo biloba extract (batch number: WGBEXP130918 ) identified by Agilent 1100 HPLC

**
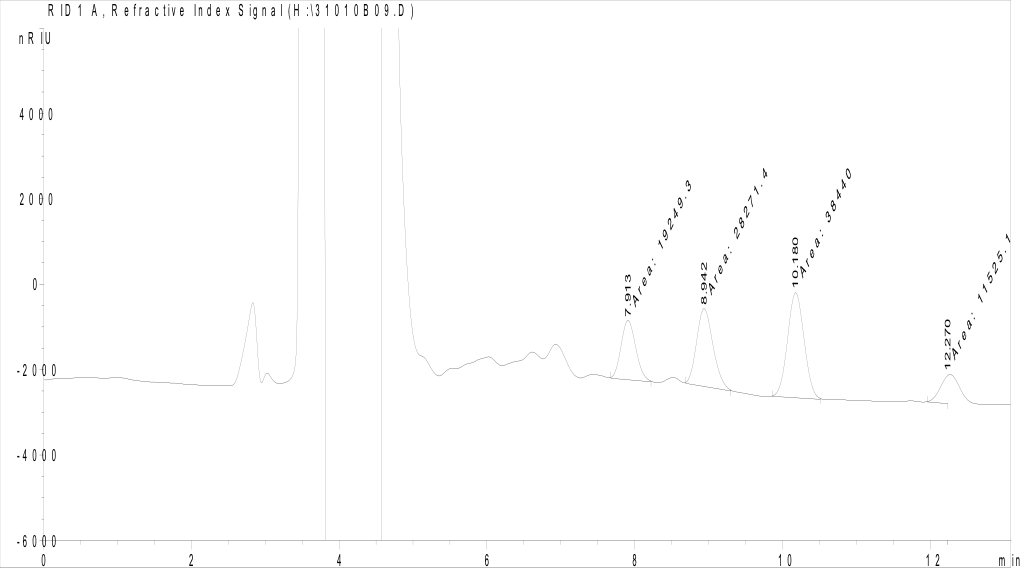
**

| Ingredient | Retention time | peak area | component |
| --- | --- | --- | --- |
| gingkolides C | 7.91 | 19249 | 1.23% |
| bilobalide | 8.94 | 18271 | 1.81% |
| gingkolides A | 10.18 | 38440 | 2.48% |
| gingkolides B | 12.27 | 11525 | 0.76% |
| total terpene lactone | ---- | ---- | 6.28% |

Figure 3: Ginkgo biloba extract (batch number: WGBEXP130918 ) certificate of anlysis

**
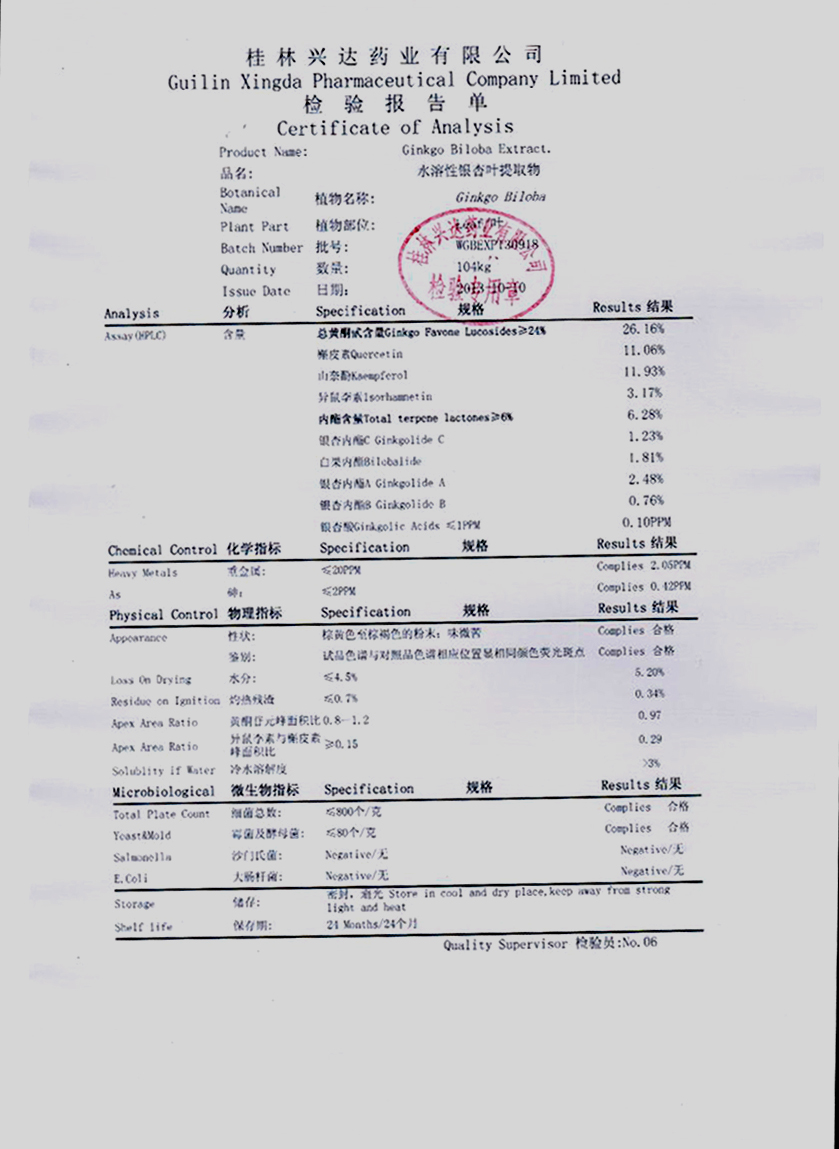
**
